# Montreal Cognitive Assessment Predicts the Short‐Term Risk of Lewy Body Disease in Isolated REM Sleep Behavior Disorder with Reduced MIBG Scintigraphy

**DOI:** 10.1002/mdc3.13569

**Published:** 2022-10-23

**Authors:** Masayuki Miyamoto, Tomoyuki Miyamoto

**Affiliations:** ^1^ School of Nursing Dokkyo Medical University Mibu Tochigi Japan; ^2^ Department of Neurology, Center of Sleep Medicine Dokkyo Medical University Mibu Tochigi Japan; ^3^ Department of Neurology Dokkyo Medical University Saitama Medical Center Koshigaya Saitama Japan

**Keywords:** dementia with Lewy bodies, mild cognitive impairment, MoCA, Parkinson's disease, phenoconversion, REM sleep behavior disorder

## Abstract

**Background:**

Long‐term follow‐up of isolated rapid eye movement (REM) sleep behavior disorder (IRBD) patients reveals a high risk of α‐synucleinopathies.

**Objective:**

We explored the early clinical predictive factors of phenoconversion from IRBD to Parkinson's disease (PD) or dementia with Lewy bodies (DLB).

**Methods:**

We assessed baseline office‐based cognitive test scores (Montreal Cognitive Assessment [MoCA‐J], Mini‐Mental State Examination [MMSE], and Frontal Assessment Battery [FAB]), motor function, and olfactory function in 36 consecutive polysomnography (PSG)‐confirmed IRBD patients with reduced metaiodobenzylguanidine (MIBG) accumulation. PD or DLB was confirmed by medical chart review retrospectively.

**Results:**

Of 36 IRBD patients, 19 (n = 19, 52.8%) with abnormal MoCA‐J score (< 26) had significantly lower scores in trail making B, phonetic verbal fluency sub‐items in the executive domain, and in delayed recall in the memory domain. In total, 12 (33.3%) patients developed PD or DLB; seven of 12 patients (58.3%) developed DLB at a mean follow‐up period of 6.8 years. In the normal MoCA‐J group (n = 17, 47.2%), two patients developed PD, but none developed dementia. Furthermore, in the abnormal MoCA‐J group, seven patients developed DLB and three developed PD without dementia. The phenoconverter group had significantly lower scores in delayed recall in the memory domain compared to the disease‐free group. Cox hazard analysis showed that MoCA‐J was superior to MMSE.

**Conclusions:**

Among IRBD patients with reduced cardiac MIBG accumulation, MoCA‐J score of <26 (Mild Cognitive Impairment‐Lewy body) and a low sub‐item score for delayed recall predicted short‐term progression to probable DLB.

Rapid eye movement (REM) sleep behavior disorder (RBD) is a REM parasomnia characterized by dream enactment behavior and REM sleep without atonia (RWA) on video‐polysomnography (video‐PSG).^1^ Idiopathic/isolated RBD (IRBD), which develops in middle or old age, suggests the existence of α‐synuclein pathology in the brain.^2^, ^3^ Long‐term follow‐up of IRBD patients has revealed high risk of α‐synucleinopathies, including Parkinson's disease (PD) or dementia with Lewy bodies (DLB) (eg, Lewy body diseases [LBD]) or multiple system atrophy.^4^, ^5^, ^6^, ^7^ However, the factors leading to increased short‐term risk of developing neurodegenerative synucleinopathies are not well‐known. Early prediction of the onset of α‐synucleinopathies in high‐risk patients is important for early interventions and disease modification therapy. Recently, the prodromal DLB diagnostic study group proposed operationalized criteria for Mild Cognitive Impairment‐ Lewy body (MCI‐LB), and ^123^I‐metaiodobenzylguanidine (^123^I‐MIBG) cardiac scintigraphy was positioned as one of the proposed biomarkers for MCI‐LB diagnosis for research.^8^

Recent reports indicate that Montreal Cognitive Assessment (MoCA) is superior to Mini‐Mental State Examination (MMSE) for the evaluation of cognitive function, particularly for the detection of MCI.^9^, ^10^ MoCA is a good measure of cognitive function because of the lack of a ceiling effect and good ability to detect cognitive heterogeneity.^10^ However, an international study of the prediction of IRBD progression showed that the combined use of MMSE and MoCA as a brief office‐based cognitive test had a hazard ratio (HR) of 1.55 (1.15–2.11), whereas MoCA alone had a non‐significant HR of 1.47 (0.93–2.32), for IRBD progression.^7^ The Japanese version of MoCA (MoCA‐J) is more useful and sensitive to predict or detect progression from IRBD to dementia, because it can effectively identify abnormal findings that are common to cognitive impairment in PD or DLB.^9^, ^10^, ^11^

In this study, we aimed to evaluate the early clinical predictive factors of disease conversion from the group defined as MCI‐LB among IRBD patients.

## Methods

The study included 36 consecutive IRBD patients who visited the sleep disorder clinic at the Dokkyo Medical University Hospital and underwent evaluation of cognitive, motor, and olfactory functions between May and November 2011 (baseline assessment in 2011). The study included 30 males (83.3%) and 6 females, with a mean age of 68.4 ± 6.2 years and number of years of education of 12.0 ± 2.6 years. Neurological examination and brain magnetic resonance imaging (MRI)s were performed to exclude neurological diseases (normal pressure hydrocephalus and vascular diseases) based on the signs of parkinsonism and/or dementia, depression, or other psychiatric diseases with cognitive impairment.

RBD was confirmed by a medical history of dream enactment behavior and video‐PSG.^1^ We also calculated the apnea‐hypopnea index (AHI), a sleep apnea parameter. The video‐PSG was performed between March 2007 and October 2011. At the 2011 baseline assessment, cognitive function was evaluated using MMSE,^12^ MoCA‐J,^13^, ^14^ and Frontal Assessment battery (FAB).^15^ For stratification, an education‐adjusted MoCA‐J score ≤25 were defined as abnormal for MCI.^7^, ^14^ In contrast, an education‐adjusted MoCA‐J score >25 were defined as normal. The motor subset of Unified Parkinson's Disease Rating Scale (1987 UPDRS part III) was used to measure motor dysfunction.^16^ Odor identification was assessed using the 40‐item Japanese version of the University of Pennsylvania Smell Identification Test (UPSIT‐J)^17^ and the Odor Stick Identification Test for Japanese (OSIT‐J) (Daiichi Yakuhin, Tokyo, Japan).^18^ ^123^I‐MIBG cardiac scintigraphy was performed, with the lower limit of the heart‐to‐mediastinum ratio for early and delayed images in ^123^I‐MIBG set to 2.2, according to the database of the Standardization Working Group of the Japanese Society of Nuclear Medicine.^19^, ^20^ We also confirmed the status of IRBD treatment at the 2011 baseline assessment.

After baseline assessment, the RBD patients were followed systematically after every 1 to 3 months by a neurologist (M.M.) with expertise in sleep disorders and neurodegenerative diseases, including dementia. At each visit, the neurologist performed a comprehensive neurological examination and obtained detailed clinical history, including related to cognitive and motor problems. In case of appearance of symptoms or signs suggestive of cognitive or motor impairment during follow‐up, the patients were referred for more detailed assessment.

Dementia was diagnosed using the Diagnostic and Statistical Manual of Mental Disorders (5th Edition) criteria.^21^ The diagnostic criteria for PD,^22^ DLB,^23^ MCI‐LB,^8^ and MSA^24^ were used to diagnose patients with these diseases, and their findings were recorded in the medical records. The medical records were reviewed between October and December 2021 to assess the presence and type of neurodegenerative diseases identified during follow‐up and date of outcome by the neurologist (M.M.).

### Statistical Analyses

Values are presented as means, standard deviations, numbers, and percentages. Comparisons between groups were performed using the Fisher's exact test for categorical variables. Mann–Whitney *U* tests were used to compare age, education, MoCA‐J, MMSE, FAB, 1987 UPDRS part III, UPSIT, and OSIT‐J scores, heart to mediastinum ratio (H/M) on early and delayed images, AHI, and score for the 12 sub‐items of MoCA‐J. The primary outcome measure was defined as the risk of developing neurodegenerative diseases or dementia (PD or DLB), which was estimated using Kaplan–Meier survival analyses for the two groups (normal and abnormal MoCA‐J scores; normal and abnormal MMSE scores). Comparisons were assessed using log rank test, and HRs were calculated according to Cox proportional hazards analyses adjusted for age and education. The significance level was set at *P* < 0.05. The statistical analyses were performed using SPSS statistical software (version 28.0; IBM Corp., Armonk, NY) and GraphPad Prism software (9.3.1 for Mac OS; GraphPad, San Diego, CA).

## Results

### Demographic Data of IRBD Patients

Demographic data of the patients at the 2011 baseline assessment are presented in Table 1. The 2011 baseline assessment of the 36 IRBD patients showed a MoCA‐J score of 24.9 ± 3.1, MMSE score of 28.2 ± 2.1, FAB score of 15.1 ± 2.0, and the 1987 UPDRS part III score of 2.2 ± 1.8. The scores of olfactory identification tests UPSIT and OSIT‐J were 18.9 ± 4.6 and 5.4 ± 2.6, respectively. In ^123^I‐MIBG cardiac scintigraphy, the H/M ratios for the early and delayed images were 1.75 ± 0.29 and 1.41 ± 0.23, respectively, and the delayed image had a lower H/M ratio than the reference value of 2.2 in all patients (Tables 1). RWA was detected in all 36 patients by video‐PSG. The PSG demonstrated an AHI of 11.4 ± 13.5/h.

**TABLE 1:** Demographic data of patients with isolated RBD

		MoCA >25	MoCA ≦25	*P*‐value
No. of patients, n (%)	36	17 (47.2)	19 (52.8)	–
Age at baseline examination, years	68.4 ± 6.2	67.2 ± 6.0	70.2 ± 6.2	0.196^a^
Gender, male/female	30/6	14/3	16/3	0.88^b^
Education, years	12.0 ± 2.6	12.8 ± 2.5	11.4 ± 2.5	0.114^a^
MoCA‐J total score	24.9 ± 3.1	27.5 ± 1.5	22.5 ± 2.0	**<0.001** ^a,c^
MMSE total score	28.2 ± 2.1	29.0 ± 1.5	27.5 ± 2.3	**0.042** ^a,c^
FAB total score	15.1 ± 2.0	15.8 ± 1.5	14.5 ± 2.2	0.057^a^
1987 UPDRS part ΙΙΙ total score	2.2 ± 1.8	2.1 ± 1.6	2.3 ± 2.0	0.827^a^
OSIT‐J	5.4 ± 2.6	5.2 ± 2.8	5.5 ± 2.5	0.95^a^
UPSIT Japanese version	18.9 ± 4.6	19.1 ± 4.8	18.7 ± 4.7	0.975^a^
H/M on early image	1.75 ± 0.29	1.70 ± 0.24	1.80 ± 0.32	0.285^a^
H/M on early image <2.2	33 (91.7)	16 (94.1)	17 (89.5)	
H/M on delayed image	1.41 ± 0.23	1.40 ± 0.22	1.41 ± 0.25	0.925^a^
H/M on delayed image <2.2	36 (100)	17 (100)	19 (100)	
AHI, per hour	11.4 ± 13.5	12.7 ± 14.1	10.3 ± 13.2	0.379^a^

Value, mean ± standard deviation. (), %. *P*‐value: ^a^Mann–Whitney *U* test; ^b^Fisher's exact test; ^c^Bold values denote statistical significance at the *P* < 0.05 level. Abbreviations; AHI, apnea hypopnea index; FAB, Frontal Assessment Battery; H/M, heart‐to‐mediastinum ratio; RBD; isolated rapid eye movement sleep behavior disorder; MMSE, Mini‐Mental State Examination; MoCA‐J, Montreal Cognitive Assessment‐Japanese version; OSIT‐J, Odor Stick Identification Test for Japanese; UPDRS, Unified Parkinson's Disease Rating Scale; UPSIT, University of Pennsylvania. Smell Identification Test.

At the time of the 2011 baseline assessment, 30 of 36 patients (83.3%) were being treated with drugs for RBD, including clonazepam (n = 29) and Yi‐Gan‐San (n = 8). In addition, five patients received continuous positive airway pressure therapy for coexisting sleep apnea.

Of 36 IRBD patients, 19 (52.8%) had abnormal MoCA‐J score (ie, diagnostic of MCI‐LB), whereas 11 (30.6%) had abnormal MMSE score. Moreover, of 25 patients with normal MMSE, 11 (44.0%) had abnormal MoCA‐J score. We compared patients with MoCA‐J score >25 (17 patients) and ≤25 (19 patients), according to the cutoff value of MoCA‐J score. Patients with MoCA‐J score >25 and ≤25 had ages of 67.2 ± 6.0 and 70.2 ± 6.2 years, respectively, and had received education for 12.8 ± 2.5 and 11.4 ± 2.5 years, respectively; there was no significant difference between the groups. A comparison between patients with MoCA‐J score >25 and ≤25 showed significant differences in the MoCA‐J score (27.5 ± 1.5 and 22.5 ± 2.0 points, respectively) and MMSE score (29.0 ± 1.5 and 27.5 ± 2.3 points, respectively). The FAB total score, 1987 UPDRS part III score, olfactory identification ability (OSIT‐J and UPSIT scores), cardiac sympathetic nerve function (H/M ratio in early and delayed images), and AHI did not show any significant difference between the two groups (Table 1).

### Score for the 12 Sub‐Items of MoCA‐J in IRBD Patients

Table 2 shows the score for the 12 sub‐items of MoCA‐J in IRBD patients. A comparison of the MoCA‐J sub‐items between the 17 patients with normal MoCA‐J score and 19 patients with abnormal MoCA‐J score showed that the abnormal MoCA‐J score group had significantly lower scores in trail making B and phonetic verbal fluency in the executive domain, and delayed recall in the memory domain. A comparison of the MoCA‐J sub‐item scores between the 24 patients who remained disease‐free and 12 patients who phenoconverted showed that the phenoconverter group had significantly lower scores in delayed recall in the memory domain (Table 3).

**TABLE 2:** Scores for the 12 sub‐items of MoCA‐J in IRBD patients

Domain Sub‐item (points)		MoCA >25	MoCA ≤25	*P*‐value
No. of patients, n (%)	36	17 (47.2)	19 (52.8)	
Visuospatial				
Copy cube (1)	0.5 ± 0.5	0.6 ± 0.5	0.4 ± 0.5	0.397
Draw clock (3)	2.8 ± 0.4	2.9 ± 0.3	2.6 ± 0.5	0.208
Executive				
Trail making B (1)	0.8 ± 0.4	1.0 ± 0.0	0.5 ± 0.5	**0.015** ^a^
Phonemic verbal fluency (1)	0.5 ± 0.5	0.9 ± 0.3	0.2 ± 0.4	**<0.001** ^a^
Verbal abstraction (2)	1.8 ± 0.5	1.9 ± 0.2	1.6 ± 0.6	0.186
Attention				
Digit span (2)	1.8 ± 0.5	2.0 ± 0.0	1.6 ± 0.6	0.061
Target tapping (1)	1.0 ± 0.2	1.0 ± 0.0	0.9 ± 0.2	0.802
Serial 7 subtraction (3)	2.8 ± 0.4	2.9 ± 0.3	2.7 ± 0.5	0.471
Memory				
Delayed recall (5)	2.5 ± 1.7	3.6 ± 1.2	1.5 ± 1.5	**<0.001** ^a^
Language				
Naming (3)	2.9 ± 0.2	3.0 ± 0.0	2.9 ± 0.3	0.594
Repeat sentences (2)	1.1 ± 0.6	1.3 ± 0.6	0.9 ± 0.6	0.093
Orientation				
Orientation (6)	5.8 ± 0.4	5.8 ± 0.4	5.7 ± 0.5	0.661
Education				
If education ≤12 years, add 1	0.7 ± 0.5	0.6 ± 0.5	0.8 ± 0.4	0.196
Total	24.9 ± 3.1	27.5 ± 1.5	22.5 ± 2.0	

Value, mean ± standard deviation. (), %. *P*‐value, Mann–Whitney *U*‐test; ^a^Bold values denote statistical significance at the *P* < 0.05 level. Abbreviations, IRBD; isolated rapid eye movement sleep behavior disorder; MoCA‐J, Montreal Cognitive Assessment‐Japanese version.

**TABLE 3:** Scores for the 12 sub‐items of MoCA‐J in IRBD patients

Domain	Sub‐item (points)		Remained disease‐free	Phenoconverted	*P*‐value
No. of patients, n (%)		36	24 (66.7)	12 (33.3)	
Visuospatial	Copy cube (1)	0.5 ± 0.5	0.5 ± 0.5	0.5 ± 0.5	1.00
	Draw clock (3)	2.8 ± 0.4	2.8 ± 0.4	2.7 ± 0.5	0.56
Executive	Trail making B (1)	0.8 ± 0.4	0.8 ± 0.4	0.6 ± 0.5	0.24
	Phonemic verbal fluency (1)	0.5 ± 0.5	0.6 ± 0.5	0.3 ± 0.5	0.07
	Verbal abstraction (2)	1.8 ± 0.5	1.7 ± 0.6	1.9 ± 0.3	0.42
Attention	Digit span (2)	1.8 ± 0.5	1.9 ± 0.3	1.5 ± 0.7	0.10
	Target tapping (1)	1.0 ± 0.2	1.0 ± 0.0	0.9 ± 0.3	0.70
	Serial 7 subtraction (3)	2.8 ± 0.4	2.9 ± 0.3	2.6 ± 0.5	0.11
Memory	Delayed recall (5)	2.5 ± 1.7	3.1 ± 1.6	1.4 ± 1.3	**0.00** ^a^
Language	Naming (3)	2.9 ± 0.2	3.0 ± 0.2	2.9 ± 0.3	0.86
	Repeat sentences (2)	1.1 ± 0.6	1.2 ± 0.6	0.8 ± 0.6	0.14
Orientation	Orientation (6)	5.8 ± 0.4	5.8 ± 0.4	5.7 ± 0.5	0.44
Education	If education ≤12 years, add 1	0.7 ± 0.5	0.8 ± 0.4	0.7 ± 0.5	0.07
Total		24.9 ± 3.1	26.1 ± 2.6	22.4 ± 2.5	

Value, mean ± standard deviation, (), % and ^a^Bold values denote statistical significance at the *P* < 0.05 level.

### Follow‐Up and Development of Neurodegenerative Diseases

The interval between estimated RBD onset and baseline assessment was 16.7 ± 8.1 years, whereas the interval from definitive RBD diagnosis to the 2011 baseline assessment was 2.1 ± 1.4 years for the 36 patients (Table 4).

**TABLE 4:** Demographic data and MoCA‐J and MMSE scores of patients with isolated RBD

MoCA	MMSE	Absolute difference	Interval between estimated RBD onset to baseline assessment, years	Interval between RBD PSG diagnosis and diagnosis or follow‐up, years	Interval between baseline assessment and diagnosis or follow‐up, years	Baseline diagnosis	Outcome
31	30	1	18.9	0.2	0.2		Disease‐free
30	30	0	47.0	10.5	10.4		Disease‐free
29	30	–1	16.6	10.2	10.3		Disease‐free
28	27	1	15.4	12.0	10.5		Disease‐free
28	30	−2	20.3	13.4	10.5		Disease‐free
28	30	–2	16.7	7.2	4.8		Disease‐free
28	25	3	4.2	1.0	1.1		Disease‐free
28	28	0	11.5	8.6	4.0		Disease‐free
27	30	−3	13.4	6.2	3.6		Disease‐free
27	30	−3	19.3	11.2	10.5		Disease‐free
27	30	−3	17.6	12.3	10.3		Disease‐free
27	30	−3	17.3	8.6	7.7		Disease‐free
26	27	−1	9.8	7.2	4.6		PD
26	30	−4	20.2	13.0	10.2		PD
26	29	−3	17.7	12.2	10.4		Disease‐free
26	29	−3	14.7	12.5	10.3		Disease‐free
26	28	−2	13.7	9.9	6.5		Disease‐free
25	24	1	14.4	12.8	10.4	pMCI‐LB	Disease‐free
25	30	−5	13.3	10.1	10.2	pMCI‐LB	Disease‐free
24	29	−5	26.9	11.7	7.5	pMCI‐LB	DLB
24	29	−5	14.3	10.7	7.9	pMCI‐LB	Disease‐free
24	30	−6	14.4	1.7	0.0	pMCI‐LB	Disease‐free
24	28	−4	40.6	9.8	6.3	pMCI‐LB	PD
24	26	−2	22.2	13.5	10.4	pMCI‐LB	Disease‐free
24	28	−4	6.0	3.1	1.7	pMCI‐LB	Disease‐free
23	24	−1	9.3	6.0	5.9	pMCI‐LB	DLB
23	30	−7	17.1	8.6	4.8	pMCI‐LB	DLB
23	29	−6	14.1	13.0	10.0	pMCI‐LB	Disease‐free
22	25	−3	6.2	5.8	2.0	pMCI‐LB	PD
22	30	−8	14.4	7.0	7.1	pMCI‐LB	DLB
21	24	−3	14.6	9.5	5.9	pMCI‐LB	DLB
21	25	−4	16.3	0.6	0.6	pMCI‐LB	DLB
21	27	−6	11.8	8.9	4.7	pMCI‐LB	Disease‐free
21	29	−8	15.3	13.2	10.0	pMCI‐LB	Disease‐free
19	26	−7	20.5	6.1	4.2	pMCI‐LB	DLB
18	30	−12	14.0	10.8	7.6	pMCI‐LB	PD
mean 24.9	mean 28.2	mean −3.3	mean 16.7	mean 8.9	mean 6.8		
SD 3.1	SD 2.1	SD 3.0	SD 8.1	SD 3.8	SD 3.5		

Abbreviations: pMCI‐LB, probable mild cognitive impairment‐Lewy body; MMSE, Mini‐Mental State Examination; MoCA‐J, Montreal Cognitive Assessment‐Japanese version; RBD; rapid eye movement sleep behavior disorder; PSG, polysomnography; SD, standard deviation.

**FIG. 1:**
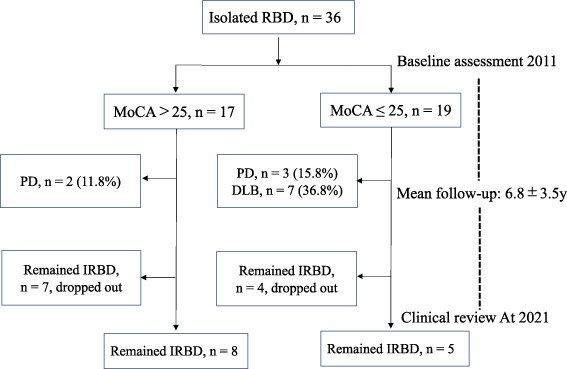
Flowchart of enrollment, follow‐up, and retrospective data collection of patients with isolated rapid eye movement disorder (RBD) between May and November 2011. PSG, polysomnography; PD, Parkinson's disease; pDLB, probable dementia with Lewy bodies; MSA, multiple system atrophy.

From the time of the 2021 outcome study, the follow‐up period for all patients was 6.8 ± 3.5 years. Of 36 IRBD patients, 12 (33.3%) developed LBD, and seven of 12 (58.3%) had DLB. Among the patients with normal MoCA‐J score (n = 17, 47.2%), two developed PD. Moreover, among patients with abnormal MoCA‐J score (n = 19, 52.8%), three (15.8%) developed PD and seven (36.8%) developed DLB (Fig. 1). The disease‐free group (n = 24, follow‐up period: 7.3 ± 3.8 years) and phenoconverted group (n = 12, follow‐up period: 5.6 ± 2.6 years) had total MoCA‐J scores of 26.1 ± 2.6 and 22.4 ± 2.5, respectively, which were significantly different between the groups (Figure S1). However, the other parameters, including MMSE score, did not differ significantly between the groups (Table 5).

**TABLE 5:** Demographic data of patients with in IRBD patients (n = 36)

	Remained disease‐free	Phenoconverted	*P*‐value
No. of patients, n (%)	24 (66.7)	12 (33.3)	
Age at baseline assessment, years	68.6 ± 6.0	69.2 ± 6.7	0.960^a^
Gender(M/F)	19/5	11/1	0.64^b^
Education, y	12.0 ± 2.4	12.2 ± 3.0	0.934^a^
Interval between baseline assessment and follow‐up, years	7.3 ± 3.8	5.6 ± 2.6	
	Median: 10.0, 0.0–10.5	Median: 5.9, 0.6–10.2	
MoCA total score	26.1 ± 2.6	22.4 ± 2.5	**<0.001** ^c^
MMSE total score	28.7 ± 1.7	27.3 ± 2.5	0.156^a^
FAB total score	15.4 ± 1.7	14.7 ± 2.5	0.562^a^
1987 UPDRS part III total score	2.5 ± 1.9	1.6 ± 1.5	0.146^a^
OSIT‐J	4.9 ± 2.6	6.4 ± 2.4	0.166^a^
UPSIT Japanese version	18.7 ± 5.0	19.3 ± 4.0	0.830^a^
H/M on early image	1.73 ± 0.24	1.81 ± 0.37	0.518^a^
H/M on early image <2.2	23 (95.8)	10 (83.3)	
H/M on delayed image	1.39 ± 0.21	1.46 ± 0.28	0.655^a^
H/M on delayed image <2.2	24 (100)	12 (100)	
AHI, per hour	12.0 ± 14.3	10.4 ± 12.2	0.608^a^

Value, mean ± standard deviation. (), %. *P*‐value: ^a^Mann–Whitney *U* test; ^b^Fisher's exact test; ^c^Bold values denote statistical significance at the *P* < 0.05 level. Abbreviations; AHI, apnea‐hypopnea index; FAB, Frontal Assessment Battery; H/M, heart‐to‐mediastinum ratio; IRBD; isolated rapid eye movement sleep behavior disorder; MMSE, Mini‐Mental State Examination; MoCA‐J, Montreal Cognitive Assessment‐Japanese version; OSIT‐J, Odor Stick Identification Test for Japanese; UPDRS, Unified Parkinson's Disease Rating Scale; UPSIT, University of Pennsylvania. Smell Identification Test.

### Risk of Neurodegenerative Diseases and Dementia

**FIG. 2:**
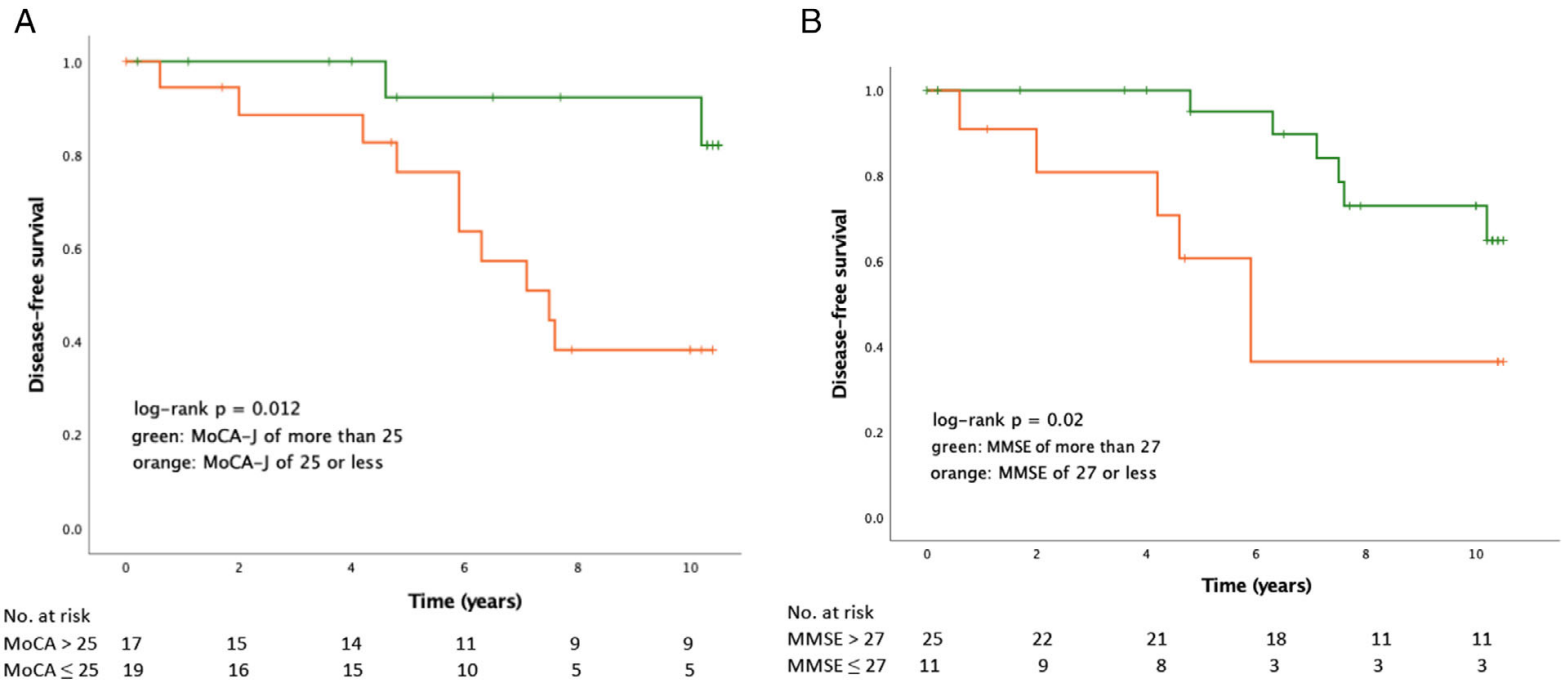
**(A**) Kaplan–Meier curves using the time of the Montreal Cognitive Assessment (MoCA) administration as the time of origin. Rates of neurological disease‐free survival according to the time of MoCA‐J score in patients with MoCA‐J score >25 (green) and MoCA‐J score ≤25 (orange) at baseline. (**B**) Kaplan–Meier curves using the time of Mini‐Mental State Examination (MMSE) administration as the time of origin. Rates of neurological disease‐free survival according to the time of MMSE score in patients with MMSE score >27 (green) and MMSE ≤27 (orange) at baseline.

Based on the MoCA‐J and MMSE scores at the time of the 2011 baseline assessment, the patients were divided into normal and abnormal MoCA‐J groups, and normal and abnormal MMSE groups, for Kaplan–Meier curve analysis. The log rank test showed that the risk of developing LBD was significantly higher in both abnormal MoCA‐J and abnormal MMSE groups (Figs. 2A,B).

In Cox hazard analysis (adjusted for age and educational history), MoCA‐J score ≤25 and MMSE score ≤27 had HRs of 5.804 (*P* = 0.026) and 4.569 (*P* = 0.025), respectively, for the development of LBD. FAB score ≤12, UPDRS part III score >3, UPSIT score ≤18, and OSIT score ≤4 were not associated with an increased risk for developing LBD after adjustment for covariates (Table 6).

**TABLE 6:** Comparison of baseline assessments in patients who developed neurodegenerative disorders and those that remained disease‐free (adjusted for age and education)

	Hazard ratio	95% CI of ratio	*P*‐value
MoCA ≤25	5.804^a^	1.239–27.186	**0.026** ^a^
MMSE ≤27	4.569^a^	1.213–17.208	**0.025** ^a^
FAB ≤12	0.533	0.216–1.315	0.172
UPDRS part III >3	0.954	0.433–2.100	0.906
UPSIT ≤18	0.856	0.474–1.545	0.606
OSIT ≤4	1.569	0.731–3.371	0.248

^a^Bold values denote statistical significance at the *P* < 0.05 level. Abbreviations; CI, confidence interval; FAB, Frontal Assessment Battery; IRBD, isolated rapid eye movement sleep behavior disorder; MMSE, Mini‐Mental State Examination; MoCA‐J, Montreal Cognitive Assessment‐Japanese version; OSIT‐J, Odor Stick Identification Test for Japanese; RBD, REM sleep behavior disorder; UPDRS III, Unified Parkinson's Disease Rating Scale part III; UPSIT, University of Pennsylvania Smell Identification Test.

In the normal MoCA‐J score group, the estimated risks of phenoconversion were 7.7% at 4.6 years and 17.9% at 10.2 years. In comparison, in the abnormal MoCA‐J group, the estimated risks of phenoconversion were 23.7% at 4.8 years and 49.1% at 7.1 years. Lower MoCA‐J score predicts short‐term risk of LBD.

## Discussion

In this study, we evaluated the early predictive factors for disease conversion from IRBD to LBD. In all IRBD patients, the mean MMSE total score was within the normal range, whereas the mean MoCA‐J total score was abnormal. This suggests that MoCA‐J is superior to MMSE for detecting subclinical cognitive decline in IRBD patients. Gagnon et al^25^ found that patients with IRBD and PD with RBD had a higher coexistence rate of MCI than healthy individuals and PD patients without RBD. In addition, RBD was a risk factor for MCI. Furthermore, they reported that MoCA was superior to MMSE for the detection of cognitive decline in RBD patients,^11^ which may be because of the difficulty of the task content (repeating sentences and delayed recall) in MoCA compared to MMSE, and because the neuropsychological symptoms observed in PD and DLB are not assessed in MMSE.

The neuropsychological symptoms observed in DLB are also observed in IRBD patients. Ferini‐Strambi et al^26^ and Terzaghi et al^27^ reported visuospatial constructional dysfunction and altered visuospatial learning in these patients. Massicotte‐Marquez et al^28^ reported a decline in attention, executive function, and verbal memory. In our study, in the group of patients with abnormal MoCA‐J score, the scores were low in the executive domain (trail making test B and phonetic verbal fluency) and memory domain (delayed recall) among the MoCA‐J sub‐items. Fantini et al^29^ performed a prospective study with baseline and 2‐year follow‐up assessment of cognitive function in IRBD patients. Although no patient developed dementia, the decline observed in some tests involving the memory and visuo‐constructional domains in IRBD suggests the presence of an underlying evolving neurodegenerative process. Génier Marchand et al^30^ reported that attention and executive function testing had high sensitivity to predict DLB in RBD patients. In addition, they^31^ reported that prodromal DLB is detectible up to 6 years before disease onset, and verbal fluency (semantic) and verbal episodic learning tests are most useful for monitoring the changes over time. In our study, as a result of longitudinal observation of IRBD patients and retrospective evaluation of patients who developed LBD, there were no patients who developed dementia in the normal baseline MoCA‐J score group. In the abnormal MoCA‐J score group, patients with low delayed recall score were found to be at high risk of developing probable DLB. When the background factors were compared between disease‐free and phenoconverted patients, there were no significant differences in terms of age, educational history, UPDRS part III score, olfactory identification, cardiac sympathetic function, coexisting sleep apnea, and RBD treatment. In elderly IRBD patients with reduced MIBG scintigraphy, cognitive function tests at baseline were shown to predict the onset of probable DLB, especially in patients with MoCA‐J total score ≤25 and low score for delayed recall.

The present study has several strengths. First, IRBD patients were confirmed to have decreased accumulation by cardiac MIBG at the time of MoCA‐J assessment. Cardiac MIBG is a proposed biomarker of MCI‐LB, as defined by McKeith et al.^8^ Matsubara et al^32^ reported that abnormal ^123^I‐MIBG myocardial scintigraphy findings strongly support the presence of LBD and cardiac sympathetic denervation. Second, the diagnosis of RBD was based on a history of dream enactment behavior and RWA confirmed by PSG. Because the diagnostic criteria of DLB requires one core symptom and two suggestive biomarkers, patients with suggestive symptoms or biomarkers may have prodromal DLB or MCI‐LB.^8^ Third, our study had a relatively long mean follow‐up period of 6.8 years (up to 10 years). The bias among observers was controlled by performing a longitudinal follow‐up from the patient's first visit to the outcome by the same neurologist.

The study also had some limitations. First, the study was conducted at a single sleep medical center located in one area. Second, the sample size was small, with about 80% of the patients being males. Third, the baseline assessment was performed at an average of 2 years after the definitive diagnosis of RBD. Fourth, at the time of the baseline assessment, 29 (80.5%) patients were already taking clonazepam for dream enactment behavior. Regarding medication use as a predictive marker, we must consider the potential link between dementia and long‐term benzodiazepine use. However, our study observed no relationship between clonazepam use and increased risk of dementia, as was found in a previous study.^33^ In this study, age and educational history were included as confounding factors for predicting the onset of dementia. It is necessary to examine the effects of genetics (Apo E4), family history (PD or dementia), socioeconomic factors, lifestyle (drinking, smoking, caffeine, and exercise), lifestyle‐related diseases (hypertension, diabetes, and obesity), depression, head injury, and drugs other than those used for RBD treatment, which have been suggested to be associated with the risk of developing dementia.^34^ Finally, in this study, the relationship between MoCA scores at the 2011 baseline and at the 2021 outcomes assessment was retrospectively investigated using a chart survey. We did not follow the changes in MoCA score prospectively and were unable to detect the cognitive fluctuation assessed by MoCA score over time.

MoCA is useful for office‐based cognitive test to detect cognitive decline in PD^35^ and IRBD^11^ patients. It is important to predict the onset of LBD in patients at risk of LBD by combining the cutoff value of MoCA score with the abnormal sub‐item scores. MoCA has several advantages, it can be performed in 10 minutes^13^ in an outpatient setting; is associated with less burden on the patient and medical doctor or neuropsychologist; and can be retested. It should also be emphasized that the short‐term prediction of developing LBD in IRBD patients is useful for screening candidates for entry into clinical trials of disease modifying therapies and neuroprotective therapy.

### Conclusions

PSG‐confirmed IRBD patients with reduced cardiac MIBG accumulation, especially those with total MoCA‐J score <26 points and low score on delayed recall, have a high risk of progression to probable DLB.

## Author Roles

(1) Research project: A. Conception, B. Organization, C. Execution; (2) Statistical Analysis: A. Design, B. Execution, C. Review and Critique; (3) Manuscript Preparation: A. Writing of the First Draft, B. Review and Critique.

M.M.: 1A, 1B, 1C, 2A, 2B, 2C, 3A, 3B.

T.M.: 1A, 1B, 1C, 2A, 2B, 2C, 3A, 3B.

## Disclosures

### Ethical Compliance Statement

This study was performed as an RBD outcome survey at the Center of Sleep Medicine at the Dokkyo Medical University Hospital. It was performed in accordance with the Declaration of Helsinki, and all procedures were approved by the Ethics Review Committee of Dokkyo Medical University (R‐2‐22). Informed consent was obtained from all participants. We informed the participants about the objectives of this research project and established a procedure for them to opt out of the study, if they did not wish to participate in it.

### Funding Sources and Conflicts of Interest

There are no funders to report for this submission. The authors declare that they have no potential conflicts of interest in relation to this article.

### Financial Disclosures for Previous 12 Months

The authors have no disclosures to report.

## Supporting information


**Figure S1.** Data distribution for the Montreal Cognitive assessment (MoCA) and Mini‐Mental state Examination (MMSE) in patients with isolated rapid eye movement sleep behavior disorder.
