# Supplementary figures and images for: Montreal Cognitive Assessment Predicts the Short‐Term Risk of Lewy Body Disease in Isolated REM Sleep Behavior Disorder with Reduced MIBG Scintigraphy
Source: Mov Disord Clin Pract. 2022 Oct 23;10(1):32–41. doi: 10.1002/mdc3.13569 (PMC9847289; doi:10.1002/mdc3.13569)

Supplementary Figure

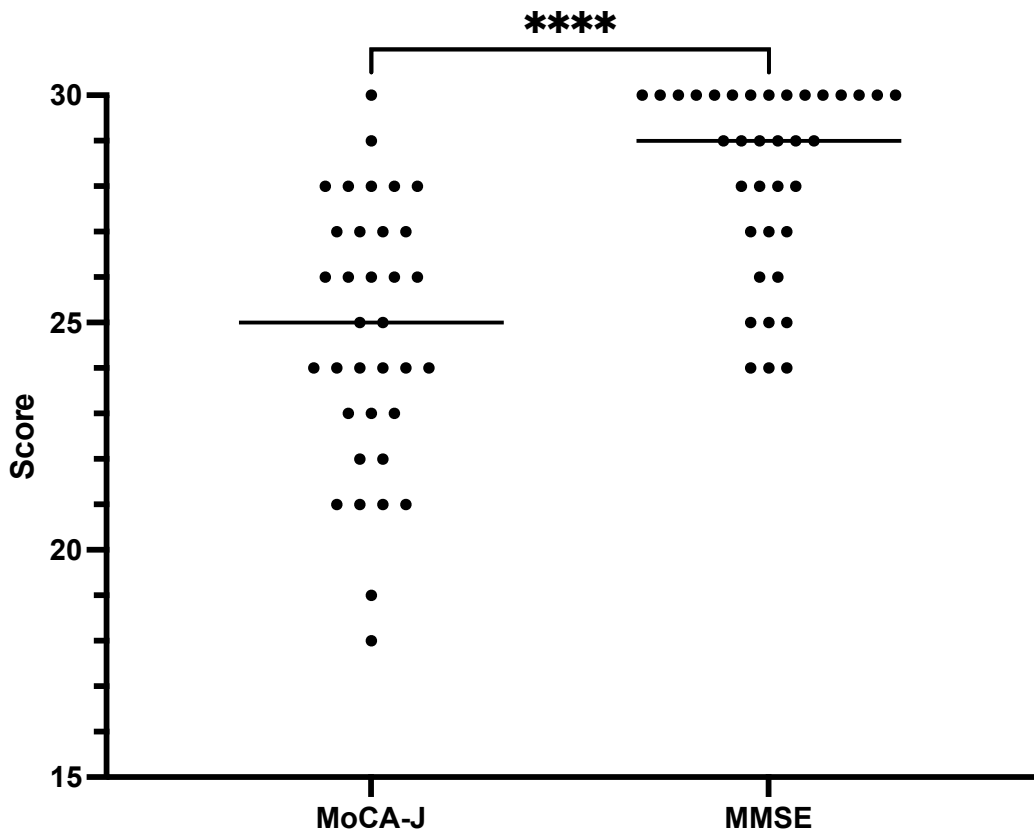

Supplement: Supplementary file 1 — Figure S1. Data distribution for the Montreal Cognitive assessment (MoCA) and Mini‐Mental state Examination (MMSE) in patients with isolated rapid eye movement sleep behavior disorder. [file MDC3-10-32-s001.pdf]
